# Exosomes rewire the cartilage microenvironment in osteoarthritis: from intercellular communication to therapeutic strategies

**DOI:** 10.1038/s41368-022-00187-z

**Published:** 2022-08-05

**Authors:** Yuangang Wu, Jiao Li, Yi Zeng, Wenchen Pu, Xiaoyu Mu, Kaibo Sun, Yong Peng, Bin Shen

**Affiliations:** 1 Orthopedic Research Institute, Department of Orthopedics, West China Hospital, Sichuan University, Chengdu, China; 2 Laboratory of Molecular Oncology, Frontiers Science Center for Disease-related Molecular Network, State Key Laboratory of Biotherapy and Cancer Center, West China Hospital, Sichuan University, Chengdu, China

**Keywords:** Regeneration, Rheumatoid arthritis

## Abstract

Osteoarthritis (OA) is a prevalent degenerative joint disease characterized by cartilage loss and accounts for a major source of pain and disability worldwide. However, effective strategies for cartilage repair are lacking, and patients with advanced OA usually need joint replacement. Better comprehending OA pathogenesis may lead to transformative therapeutics. Recently studies have reported that exosomes act as a new means of cell-to-cell communication by delivering multiple bioactive molecules to create a particular microenvironment that tunes cartilage behavior. Specifically, exosome cargos, such as noncoding RNAs (ncRNAs) and proteins, play a crucial role in OA progression by regulating the proliferation, apoptosis, autophagy, and inflammatory response of joint cells, rendering them promising candidates for OA monitoring and treatment. This review systematically summarizes the current insight regarding the biogenesis and function of exosomes and their potential as therapeutic tools targeting cell-to-cell communication in OA, suggesting new realms to improve OA management.

## Introduction

Osteoarthritis (OA) is a chronic low-degree inflammatory disease mainly characterized by progressive degeneration of articular cartilage, thickening of the subchondral bone, synovial inflammation, meniscus and ligament degeneration, and osteophyte formation.^1,2^ The well-established risk factors for OA include age, sex, obesity, trauma, metabolism, and joint biomechanics.^3–5^ The chronic pain and dysfunction caused by OA affect over 250 million people worldwide,^2^ which severely reduces the life quality of individuals and represents a considerable socioeconomic burden.^6^ Currently, drug therapy serves as a fundamental strategy in the overall treatment of OA.^7,8^ Throughout the whole treatment process, most patients need short-term or long-term medication,^7–9^ including nonsteroidal anti-inflammatory drugs (NSAIDs), opioids, and drugs for intra-articular injection (e.g., hyaluronic acid and glucocorticoid). However, the current drug treatment of OA suggested by international guidelines is merely aimed at remission of disease symptoms, without substantial interruption of the destructive process or restoration of lesioned cartilage in OA.^8–10^ As for patients with end-stage OA, joint arthroplasty surgery represents a prevalent treatment modality, although sometimes the functional outcome can be unsatisfactory. Moreover, joint replacement requires more revision surgery in the case of complications such as infection and prosthetic fracture.^11,12^ Therefore, it is essential to clarify the molecular mechanisms underlying OA occurrence and progression to facilitate new therapies for future clinical needs.

**Fig. 1:**
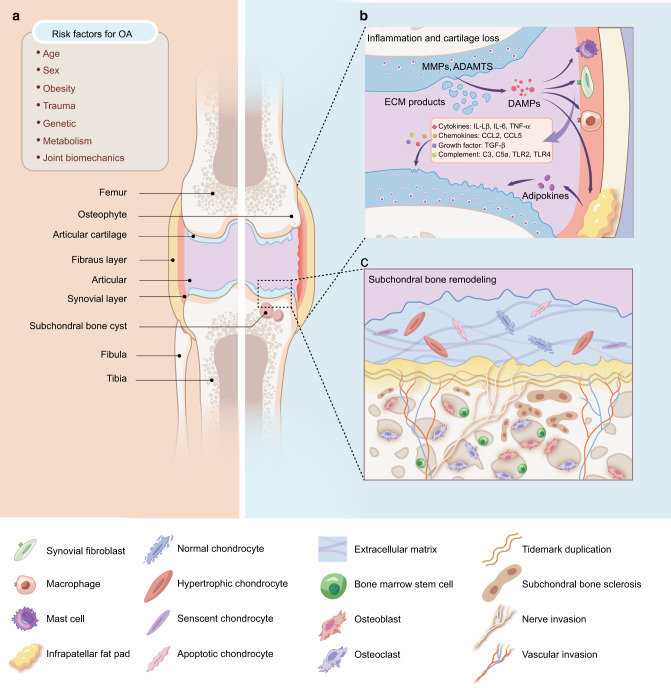
The inflammatory network and pathobiology in OA. **a** Risk factors for OA. Evidence suggests that specific systemic risk factors (e.g., age, obesity, and sex) and mechanical factors (e.g., trauma and joint biomechanics) are capable of causing cartilage damage in OA. **b** Inflammatory network in OA. The degradation products of cartilage and extracellular matrix (ECM) components are released into the joint cavity as damage-associated molecular patterns (DAMPs), which ultimately stimulate synovial cells and macrophages to secrete different types of inflammatory mediators, such as cytokines, chemokines, and complement. These molecules further lead to loss of chondrocyte phenotype and cartilage damage. Notably, the fat pad around the joints has been identified as another source of proinflammatory cytokines, especially adipokines, which may also trigger a catabolic response in chondrocytes. **c** Changes of osteochondral structure in OA. With the progression of OA, hypertrophy, senscent, and apoptotic chondrocytes increase, resulting in ECM catabolism as well as cracks and stratification appearing on the surface of cartilage. In the subchondral bone region, subchondral bone thickness gradually increases and calcified cartilage extends into articular cartilage, with duplication of the tidemark and invasion of vascular and nerve invasion into the cartilage region. Overall, these changes in the cartilage microenvironment can accelerate the degeneration progression of cartilage. MMPs matrix metalloproteinases, ADAMTSs a disintegrin and metalloproteinase with thrombospondin motifs, ECM extracellular matrix, DAMPs damage-associated molecular patterns, IL-1β interleukin-1beta, IL-6 interleukin-6, TNF-α tumor necrosis factor-alpha, CCL2 chemokine (C-C motif) ligand 2, CCL5 chemokine (C-C motif) ligand 5, TGF-β transforming growth factor-beta, TLR2 Toll-like receptor 2, TLR4 Toll-like receptor 4

Joint cartilage tissue is in a complex microenvironment that contains not only chondrocytes but also a variety of non-chondrocyte types, such as adipocytes, synovial cells, mesenchymal stem cells (MSCs), endothelial cells, and immune cells.^1,3,5^ These cellular components can crosstalk with each other by secreting a variety of metabolic factors and inflammatory factors through paracrine, autocrine, and endocrine pathways and jointly maintain articular cartilage homeostasis, which is, however, heavily disrupted in OA. Accumulating evidence suggests that altered communication between chondrocytes and the surrounding tissues may directly or indirectly affect the progression of OA^1,13^ (Fig. 1). Upon exposure to risk factors that promote joint vulnerability, dysfunctional chondrocytes release excessive protease matrix-degrading enzymes, such as matrix metalloproteinases (MMPs) and a disintegrin and metalloproteinase with thrombospondin motifs (ADAMTS), leading to the degradation of extracellular matrix (ECM).^14^ These degradative products are released into the synovial fluid, where they act as damage-associated molecular patterns (DAMPs) to trigger the inflammatory response of adjacent synovial cells (e.g., synovial fibroblasts, macrophages, and mast cells). Subsequently, OA-related immune components, including proinflammatory cytokines (e.g., interleukin-1β (IL-1β), interleukin-6 (IL-6), and tumor necrosis factor-alpha (TNF-α)), growth factors (e.g., transforming growth factor-beta, (TGF-β)), chemokines and adipokines, are aggregated and further promote the activity of MMPs and ADAMTSs, initiating vicious feedback of local tissue damage and low-grade inflammation.^14,15^ Therefore, exploring how cells communicate within cartilage microenvironments may help to unveil the pathogenesis of OA and explore new strategies for future treatment of OA.

Recently, exosomes have emerged as a new medium involved in cell–cell communication.^16,17^ A variety of cellular components, in particular noncoding RNAs (ncRNAs), can be preferentially capsuled into exosomes and then delivered to the surrounding or distant environment. Exosomal cargoes can regulate the biological activity of recipient cells, thus shuttling intercellular signals and triggering various physiological and pathological processes. Multiple cell lineages in the osteoarthritic joint are able to behave as active aggressors or passive responders by secreting or internalizing exosomes during disease onset and progression. Better characterization of exosomal cargoes might facilitate the identification of new indicators and inform targeted therapy for OA. In this review, we highlight the important role of exosomes in the progression of OA and discuss the prospects and challenges of their utility as clinical biomarkers or therapeutic agents for OA patients.

## The biogenesis and release of exosomes

**Fig. 2:**
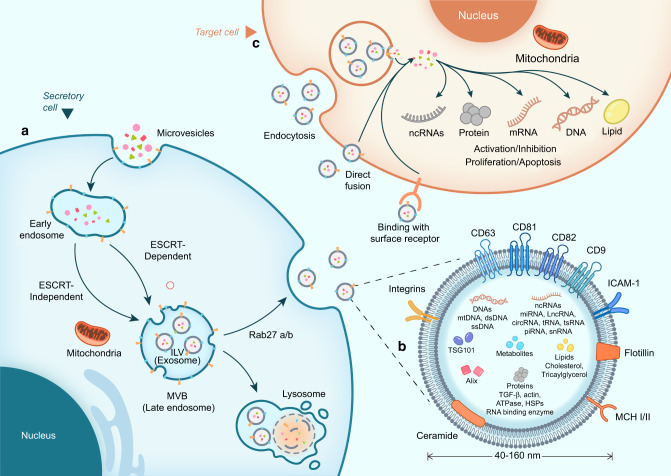
The processes of exosome biogenesis and release. **a** Biogenesis and release of exosomes. Exosome biogenesis originates from plasma membrane endocytosis, followed by maturation of early endosomes as MVBs. During this process, ILVs (exosomes) are formed within MVBs by ESCRT-dependent or ESCRT-independent pathways. In secretory cells, MVBs can be degraded by lysosomes or fused with the plasma membrane to release ILVs as exosomes with the assistance of Rab27a/b. **b** The contents of exosomes. Exosomes contain a variety of intrinsic components and variable cargoes, varying from proteins, lipids, metabolites, and DNAs to ncRNAs. **c** Interaction between exosomes and the target cells. In target cells, exosomes can directly bind with surface receptors to trigger intercellular signaling. Alternatively, exosomes can be internalized via endocytosis or direct fusion and liberate their cargoes into the target cells, thus mediating multiple physiological and pathological processes. MVBs multivesicular bodies, ILV intracavitary vesicles, ESCRT endosomal sorting complexes required for transport

Exosomes, ranging from 40 to 160 nm in diameter,^16^ are lipid-bilayer extracellular vehicles (EVs) secreted by most eukaryotic cells and abundant in various body fluids, including blood, urine, saliva, breast milk, pleural effusion, and synovial fluid^16,18–20^ (Fig. 2). Typically, exosomes contain a group of common membrane and cytoplasmic proteins, including membrane transport and fusion proteins (Rab GTPases, annexin, integrin, and fibronectin),^21,22^ tetraspanins (CD9, CD63, CD81, and CD82),^23,24^ heat shock proteins (Hsp20 and Hsp27),^25^ and lipid-related proteins.^26,27^ The biogenesis of exosomes initiates with the double invagination of the plasma membrane to generate early endosomes, which subsequently maturate to intracellular multivesicular bodies (MVBs), the intermediates within the endosomal system. During this transition, exosomes, or essentially speaking, intraluminal vesicles (ILVs), are formed within the luminal space of MVBs.^18,19^ This process involves a continuum of mechanisms and particular sorting machinery to sequester cargoes on microdomains of the limiting membranes of MVBs, followed by inward membrane budding and fission of small vesicles harboring separated cytosol. The center of these machineries is the endosomal sorting complex required for transport (ESCRT),^28,29^ which acts as a driver of membrane shaping and scission of ILVs. The ESCRT machinery is mainly composed of four distinct subcomplexes known as ESCRT-0, -I, -II, and-III.^28–30^ ESCRT-0 and ESCRT-I are involved in cargo aggregation on the microdomains of MVB membranes. ESCRT-II is required for the assembly and recruitment of the ESCRT-III subcomplex, while ESCRT-III performs the budding and fission of ILVs into the MVB lumen.^23^ The canonical ESCRT pathway can also crosstalk with the syntein/ALIX axis to manipulate cargo clustering and membrane budding, which likely depends on the ability of ALIX to bridge the ESCRT-III subunit and cargo at the neck of ILVs.^30^ Notably, exosomes are still formed even when the ESCRT complex is exhausted,^31^ suggesting an ESCRT-independent mechanism for exosome biogenesis. Wei et al^32^ demonstrated that upon epidermal growth factor (EGF) stimulation, Rab GTPase 31 (RAB31) is phosphorylated by the epidermal growth factor receptor (EGFR) and further interacts with flotillin proteins in lipid raft microdomains to drive EGFR entry into ILVs in an ESCRT-independent manner. Therefore, diverse machinery can act on the endosomal system to target and package cargoes scheduled for secretion, and their involvement and contribution also shift due to cell types and the signals or stimuli that cells receive, leading to spatiotemporal dynamics of the exosomal compositional repertoire.

Upon maturation, MVBs are basically targeted to lysosomes for degradation or alternatively transported along with microtubules to the plasma membrane for secretion, but the mechanism to balance the secretion and degradation of MVBs remains largely unknown. Once docked at the plasma membrane, the accumulated nondegradable MVBs fuse with the membrane to release ILVs as exosomes, which primarily rely on the common secretory machinery and can be promoted by several Rab family members, such as Rab27a and Rab27b.^33,34^ After secretion into the extracellular space, the double phospholipid membrane of exosomes protects its content from premature degradation during transportation and enables site-specific anchoring toward recipient cells.^35,36^ Subsequently, exosomes can interact with recipient cells in various ways, depending on cell types and the origin of exosomes.^36^ They can be maintained on the surface of recipient cells and directly activate surface receptors to elicit functional responses (e.g., antigen presentation). Alternatively, they can be internalized by recipient cells via direct fusion with the cell plasma membrane or via a variety of endocytic pathways, including clathrin-mediated endocytosis, micropinocytosis, phagocytosis, and caveolin- or lipid raft-mediated uptake.^37,38^ Following the above internalization pathways, exosomes release their contents into the cytoplasm of receptor cells and can also be degraded by lysosomes for recycling.^18,35,36^

## Function of exosomes

The unique mechanisms governing the biogenesis of exosomes confer on them distinct sets of components, varying from proteins to nucleic acids and lipids. Their surface molecules enable physical interactions with cell populations, and their onboard cargoes initiate biological responses and phenotypic changes in recipient cells. Thus, exosomes have emerged as novel vehicles for intercellular communication, allowing the dynamic exchange of material and information between cells, tissues, and organ systems. The current interest in the field has focused on exploring the biological activity of exosomes in maintaining homeostatic balance or as a driver or consequence of pathological processes.^39^

Extensive studies have shown that exosomes serve as a key medium for cell–cell communication in joint cartilage tissue and are involved in the pathogenesis of OA.^40,41^ Cartilage-derived exosomes can impinge on the biological behavior of surrounding stromal cells, ultimately creating an appropriate microenvironment for cartilage growth.^42^ At the same time, stromal cells in the cartilage microenvironment can also release exosomes carrying anabolic/catabolic and anti-/proinflammatory factors, which in turn affect chondrocyte proliferation, apoptosis, and inflammatory responses, as well as the synthesis and catabolism of the cartilage ECM.^41^ Thus, exosome-mediated complex interactions between chondrocyte and stromal cells ensure cartilage homeostasis, while the perturbation of these processes triggers different forms of imbalances that can cause inflammation and thickening of cartilage tissue, eventually initiating or accelerating the progression of OA.

## Exosomal ncRNAs in OA

**Fig. 3:**
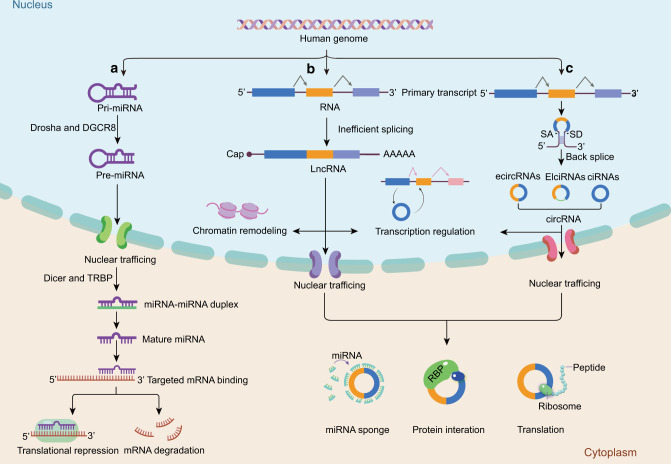
The biogenesis and functions of miRNAs, lncRNAs, and circRNAs. **a** Biogenesis and functions of miRNA. After transcription by RNA polymerase II in the nucleus, primary miRNA (pri-miRNA) is cleaved by DGCR8 and the Drosha complex into precursor-miRNA (pre-miRNA). The pre-miRNA is subsequently transported from the nucleus to the cytoplasm, where it is further processed by Dicer and TRBP to a miRNA duplex. Next, the guide strand (mature miRNA) remains to form RISC protein complexes and ultimately mediates translation repression and mRNA degradation. **b** Biogenesis and functions of lncRNA. Several primary transcripts undergo inefficient splicing to generate lncRNAs with a 5′ cap and a 3′ poly-(A) tail structure. In the nucleus, lncRNAs are associated with chromatin remodeling and gene transcription regulation. In the cytoplasm, they can exert their function by acting as miRNA sponges, binding with proteins, or encoding regulatory peptides. **c** Biogenesis and functions of circRNA. circRNA forms a closed-loop structure primarily produced from pre-mRNAs via back-splicing. Based on the original sources, circRNAs can be divided into ciRNAs, EIciRNAs, and exonic ecircRNAs. ciRNAs and EIciRNAs are involved in the transcriptional modulation of their parent genes, while ecircRNAs primarily reside in the cytoplasm and can interact with miRNAs and proteins or be translatable. DGCR8 DiGeorge syndrome critical region 8, TRBP TAR RNA-binding protein, RISC RNA-induced silencing complex, ciRNAs circular intronic RNAs, EIciRNAs exon-intron circRNAs, ecircRNAs exonic circRNAs

Comprehensive profiling of exosomes derived from joint tissues and fluids has demonstrated that they are enriched in ncRNAs^43,44^ (Fig. 3), including microRNAs (miRNAs), long noncoding RNAs (lncRNAs), and circular RNAs (circRNAs). These molecules can be preferentially loaded into exosomes and shuttled between chondrocytes or surrounding stromal cells.^45,46^ Moreover, the proportion of these cargoes varies depending on the donor cell types and OA staging, suggesting their properties to indicate disease status. Consequently, the focus on characterizing exosomal cargoes in OA is currently growing, and diverse exosomal ncRNAs have been documented to exhibit distinct expression profiles in the gain or maintenance of OA phenotypes.

miRNAs are endogenous small ncRNAs with a length of approximately 20–22 nucleotides. The biogenesis of miRNAs is precisely processed via a series of RNA polymerases, RNases, and RNA-binding proteins (RBPs).^47–49^ In many cases, miRNAs bind to the 3′ untranslated regions (UTRs) of messenger RNA (mRNA) to cause translational repression or target mRNA degradation.^47^ Abundant evidence supports the selective enrichment of miRNAs in exosomes originating from articular tissues. Importantly, exosomal miRNAs exhibit distinct patterns between OA and non-OA samples. A miRNA microarray performed on exosomes from primary chondrocytes suggested that 22 miRNAs were upregulated and 29 were downregulated in OA chondrocyte-secreted exosomes compared to the normal group.^50^ Another study examined exosomes from the synovial fluid of OA patients and healthy subjects,^51^ and the profiling of exosomal contents revealed a remarkable difference in miRNA expression, implying altered intercellular communication within the OA microenvironment.

LncRNAs are designated noncoding transcripts with a length of more than 200 nucleotides.^52,53^ Similar to mRNA processing, most lncRNAs are transcribed via polymerase II but probably undergo inefficient splicing.^54^ Based on their subcellular localization, lncRNAs can coordinate with diverse nuclear acids and proteins to regulate gene expression at multiple levels.^55–60^ The important roles of lncRNAs in OA progression have emerged. For example, lncRNA NEAT1 could inhibit chondrocyte proliferation and promote apoptosis by functioning as a sponge for miR-543.^61^ Of particular interest is the existence of certain lncRNAs in exosomes isolated from body fluids, with differential expression profiles between OA patients and healthy populations,^62^ indicating their preferential packaging into exosomes and potential involvement in OA evolution.

Unlike the typical linear ncRNAs, circRNAs form a closed-loop structure primarily produced from pre-mRNAs via back-splicing,^63,64^ endowing them with inherent resistance to exonuclease-mediated decay and thus greater stability than linear RNAs. The recent advance in RNA sequencing (RNA-seq), together with the specialized computational pipelines, has led to an explosion in circRNA characterization.^65–70^ Most circRNAs are predominantly distributed in the cytoplasm and execute their functions by acting as “miRNA sponges”, protein “decoys” or scaffolds, or by coding regulatory peptides.^69–72^ It is worth noting that circRNAs are also prevalent in various exosomes and can be transferred to recipient cells as influential messengers. The field to characterize exosomal circRNAs in OA is in infancy. A subset of exosomal circRNAs originating from human chondrocytes and MSCs actively participate in chondrogenesis and cartilage degradation.^73,74^ A future comprehensive illustration of exosome cargo will provide new insight into the role of circRNAs in OA pathology.

**Fig. 4:**
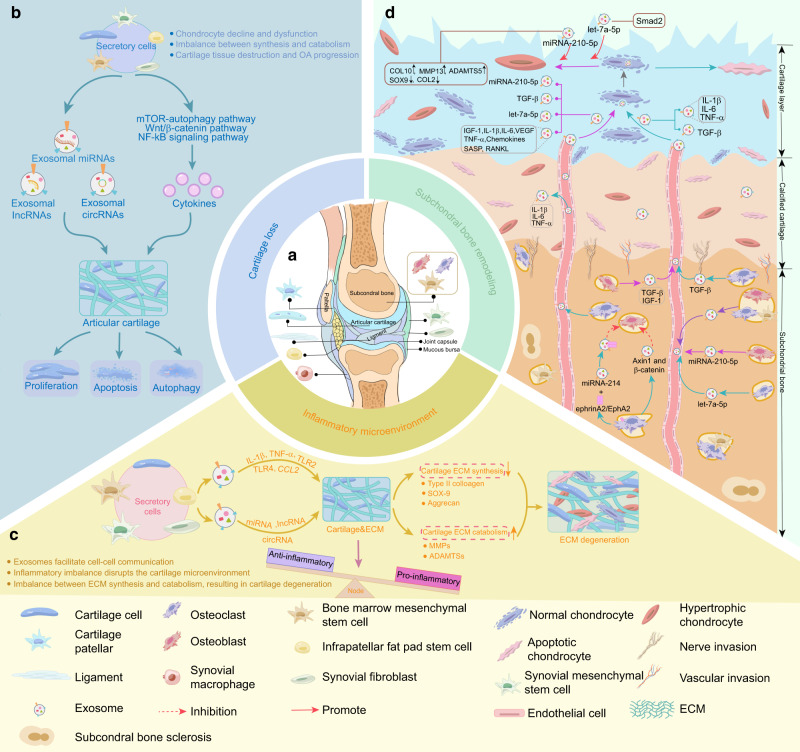
The major biological functions of different tissue-derived exosomes in OA. **a** Exosomes can be released by various periarticular cells, including chondrocytes, osteoblasts, osteoclasts, and bone marrow MSCs in subchondral bone, synovial fibroblasts, macrophages, and MSCs in the synovium and infrapatellar fat pad. **b** Exosomes can mediate cell-to-cell communication to regulate multiple phenotypes of chondrocytes, including proliferation, apoptosis, and autophagy, thus affecting cartilage damage. **c** Inflammatory factors, noncoding RNAs, and other mediators released by exosomes from different cells contribute to the remodeling of the inflammatory microenvironment and drive ECM catabolism. **d** Exosomes and cytokines produced by osteoblasts, osteoclasts, and bone marrow MSCs in subchondral bone are transported to the cartilage layer and regulate chondrocyte metabolism. In addition, exosomes secreted by osteocytes can participate in subchondral bone remodeling, leading to changes in the biomechanical properties of OA subchondral bone, ultimately causing damage to the cartilage. OA osteoarthritis, MSCs mesenchymal stem cells, IL-1beta interleukin-1β, IL-6 interleukin-6, TNF-α tumor necrosis factor-alpha, CCL2 chemokine (C-C motif) ligand 2, TLR2 Toll-like receptor 2, TLR4 Toll-like receptor 4, MMP13 matrix metalloproteinase 13, ADAMTS5 a disintegrin and metalloproteinase with thrombospondin motifs 5, ECM extracellular matrix, MMP matrix metalloproteinase 13, SOX9 SRY-box transcription factor 9, mTOR mechanistic target of rapamycin, Wnt/β-catenin Wnt-beta-catenin, NF-κB nuclear factor kappa-B, VEGF vascular endothelial growth factor, TGF-β1 transforming growth factor-β1, IGF-1 insulin-like growth factor-1, COL2 type 2 collagen, COL10 type 10 collagen, RANKL receptor activator of NF-κB-associated phenotype β1

OA is a progressive joint disorder typically driven by cartilage loss/breakdown, subchondral bone remodeling, and synovial inflammation.^1,2^ Exosomal ncRNAs from different joint cells, such as chondrocytes,^50,75^ MSCs,^76,77^ fibroblast-like synoviocytes (FLSs),^78,79^ osteoclasts^80^ and osteoblasts,^81^ alter the proliferation,^73,82^ apoptosis,^83^ inflammatory response and differentiation^84^ abilities of target cells, thus possibly disrupting joint tissue homeostasis and affecting the development of OA (Fig. 4). In the following section, we summarize the current studies on the specific roles and mechanisms of exosomal miRNAs (Table 1), lncRNAs (Table 2), and circRNAs (Table 3) in the progression of OA.

**Table 1:** The role of exosomal miRNAs in the pathogenesis of OA

miRNAs	Sample	Exosome source	Role	Reference
miR‐95‐5p	Human	Chondrocytes	Enhances chondrogenesis and prevents the development of OA by directly targeting HDAC2/8	^50^
miR-449a-5p	Human	OA chondrocytes	Inhibits ATG4B and autophagy in macrophages	^75^
miR-140-5p	Human	Synovial MSCs	Enhances the proliferation, migration of articular chondrocytes, and prevents OA in a rat model	^76^
miR-100-5p	Human	Infrapatellar fat pad MSCs	Ameliorates the OA degeneration by targeting the mTOR-autophagy pathway	^77^
miR-135b	Rats	MSCs	Promotes C5.18 cell (rat chondrocyte cell line) proliferation by regulating Sp1	^82^
miR-92a-3p	Human	MSCs	Enhances chondrogenesis and suppresses cartilage degradation by targeting WNT5A	^84^
miR‐100‐5p	Human	Human umbilical cord MSCs	Inhibits cyclic strain‐induced ROS production and apoptosis in chondrocytes by targeting NOX4	^97^
miR-126-3p	Rats	Synovial fibroblasts	Promotes chondrocyte proliferation and suppresses apoptosis by constraining chondrocyte inflammation	^113^
miR-135b	Rats	Bone marrow-derived MSCs	Attenuates cartilage injury by promoting synovial macrophage M2 polarization by targeting MAPK6	^123^
miR-136-5p	Human	Bone marrow-derived MSCs	Inhibits chondrocyte degeneration in OA by targeting ELF3	^158^
miR-9-5p	Rats	Bone marrow-derived MSCs	Alleviates OA degeneration by targeting SDC1 in an OA rat model	^159^
miR-140-5p	Human	Dental pulp stem cells	Inhibits IL-1β-induced chondrocyte apoptosis	^161^
miR-140	Rats	Dendritic cells	Alleviates OA progression in a rat model	^162^
miR-140-5p	Human	Urine-derived stem cells	Inhibits the progression of KOA by mediating VEGFA	^165^
miR-320c	Human	Bone MSCs	Promotes chondrogenic differentiation	^166^
miR-26a-5p	Human	Bone MSCs	Alleviates OA via downregulation of PTGS2	^167^
miR-372-3p	Human	OA chondrocytes	Promotes chondrocyte proliferation and inhibits apoptosis	^168^
miR-127-3p	Rats	Bone marrow-derived MSCs	Alleviates OA by regulating the CDH11-mediated Wnt/β-catenin pathway	^169^
miR-206	Mice	Bone marrow-derived MSCs	Promotes proliferation and differentiation of osteoblasts in OA by reducing ELF3	^170^
miR-147b	Human	MSCs treated with IL-1β and TNF-a	Inhibits the inflammatory response of OA SW982 cells (Synovial cell line)	^171^
miR-129-5p	Human	Synovial MSCs	Relieves IL-1β induced OA by targeting HMGB1	^172^
miR-361-5p	Human	Bone MSCs	Alleviates OA by targeting DDX20 and NF-κB signaling pathway	^173^
miR-193b-3p	Human	Plasma	As a marker to distinguish expression levels between normal and OA patients	^174^

*OA* osteoarthritis, *MSCs* mesenchymal stem cells, *HDAC* histone deacetylase, *SOX9* SRY-box transcription factor 9, *Sp1* specificity protein-1, *ATG4B* autophagy-related 4B, *VEGFA* vascular endothelial growth factor A, *MAPK6* mitogen-activated protein kinase 6, *mTOR* mechanistic target of rapamycin, *SDC1* syndecan-1, *PTGS2* prostaglandin-endoperoxide synthase 2, *CDH1* cadherin-11, *ROS* reactive oxygen species, *NOX4* NADPH oxidase 4, *HMGB1* high mobility group protein B1, *DDX20* Asp-Glu-Ala-Asp-box polypeptide 20, *NF-κB* nuclear factor kappa-B

**Table 2:** The role of exosomal lncRNAs in the pathogenesis of OA

lncRNAs	Sample	Exosome source	Role	Reference
lncRNA‑PVT1	Human	C28/I2 cells (human chondrocyte cell line)	Modulates chondrocyte viability, apoptosis, and inflammation responses by miR-93-5p/HMGB1/TLR4/NF-κB pathway	^62^
lncRNA-H19	Human	Fibroblast-like synoviocytes	Promotes chondrocyte proliferation and migration and inhibits matrix degradation in OA possibly by targeting the miR-106b-5p/TIMP2 axis	^78^
lncRNA-PCGEM1	Human	Fibroblast-like synoviocytes	Facilitates IL-1β-induced apoptosis and cartilage matrix degradation in chondrocytes by targeting the miR-142-5p/RUNX2 axis	^79^
lncRNA KLF3-AS1	Human	MSCs	Promotes chondrocyte proliferation and inhibits apoptosis via the miR-206/GIT1 axis	^83^
lncRNA-PCGEM1	Human	Synovial fluid	An indicator to distinguish early OA from late OA	^155^
lncRNA KLF3-AS1	Human	MSCs	Promotes chondrocyte proliferation and cartilage repair in OA	^175^
lncRNA-H19	Human	Umbilical cord blood MSCs	Improves pain and central sensitization of advanced OA via miRNA-29a-3p/FOS axis	^176^

*OA* osteoarthritis, *MSCs* mesenchymal stem cells, *GIT1* G-protein coupled receptor kinase interacting protein-1, *SOX9* SRY-box transcription factor 9, *LPS* lipopolysaccharide, *HMGB1* high mobility group protein B1, *TLR4* toll-like receptor 4, *NF-κB* nuclear factor kappa-B, *TIMP2* TIMP metallopeptidase inhibitor 2, *GRPR* gastrin-releasing peptide receptor, *IL-1β* interleukin-1β, *RUNX2* runt-related transcription factor 2

**Table 3:** The role of exosomal circRNAs in the pathogenesis of OA

circRNAs	Sample	Exosome source	Role	Reference
circ-BRWD1	Human	Chondrocyte cell line CHON-001 treated with IL-1β	Promotes OA progression by regulating the miR-1277/TRAF6 axis	^73^
circHIPK3	Human	MSCs	Promotes chondrocyte proliferation and migration and suppresses apoptosis via the miR-124-3p/MYH9 axis	^74^
circ_0001846	Human	Chondrocyte cell line CHON-001 treated with IL-1β	Modulates IL-1β-induced chondrocyte damage by miR-149-5p/WNT5B axis	^99^
circRNA_0001236	Human	MSCs	Alleviates cartilage degradation through the miR3677-3p/Sox9 axis	^177^
circRNA3503	Human	CircRNA3503-overexpressed synovium MSCs	Alleviates chondrocyte apoptosis and ECM imbalance by acting as sponges of miR181c-3p and let-7b-3p	^178^

*OA* osteoarthritis, *MSCs* mesenchymal stem cells, *IL-1β* interleukin-1β, *TRAF6* TNF receptor-associated factor 6, *MYH9* myosin heavy chain 9, *SOX9* SRY-box transcription factor 9, *ECM* extracellular matrix, *WNT5B* wingless-type MMTV integration site family member 5B

### Exosomal ncRNAs and cartilage loss in OA

Articular cartilage loss represents the major hallmark of OA. The maintenance of cartilage integrity relies largely on the cartilage ECM, which provides the structural basis for the functional properties of articular cartilage by formulating a dense and highly organized collagenous network mainly comprised of type II collagen, as well as proteoglycans, particularly aggrecan.^85^ As the unique cellular component of articular cartilage, chondrocytes are solely responsible for ECM synthesis. The dynamic balance between anabolic and catabolic metabolism in the ECM is disrupted in the pathological condition of OA, where the synthesis of ECM no longer compensates for the loss of matrix integrity.^14^ MMPs and ADAMTS are two main families of proteinases that lead to the degradation of ECM, in particular MMP13 and ADAMTS5,^86,87^ which can be produced by chondrocytes or other joint cell lineages and induced by cytokines such as IL-1β and TNF-α in the extracellular space. Deletion of MMP13 and ADAMTS5 in mice can effectively control the degradation of ECM and the progression of OA degeneration.^87,88^ Therefore, the underlying mechanism to trigger the imbalance of ECM metabolism is multifactorial, involving dynamic crosstalk between joint cells within the cartilage microenvironment.

Chondrocytes are sparsely embedded within the ECM and constitute 2–5% of the cartilage tissue.^89^ Although residing in a metabolically “resting” state, chondrocytes can respond to mechanical stimuli or active biomolecules in the surrounding microenvironment in a positive or negative manner, leading to variations in the phenotype and metabolism of chondrocytes themselves and eventual changes to the composition of the ECM. Notably, the interaction between chondrocytes and their surroundings can be mediated by exosomes carrying various ncRNAs.^83^

#### Exosomal ncRNAs regulate chondrocyte proliferation in OA

Cartilage degeneration in the aged joint is tightly related to the decreased number of chondrocytes, which fail to regenerate the ECM properly. In the initial stage of OA, chondrocytes manifest transiently increased proliferation ability, which is interpreted as a repair response.^89^ Exosomal ncRNAs exhibit significant roles in regulating OA chondrocyte proliferation. Wang and colleagues^82^ investigated the potential function of exosomal miRNAs in a rat model. They found that miR-135b was upregulated in rat MSC-derived exosomes upon transforming growth factor-β1 (TGF-β1) stimulation and promoted the proliferation of rat chondrocyte C5.18 cells. Mechanistically, miR-135b negatively regulates the expression of Sp1, a transcription factor that inhibits the cell cycle by activating p15^INK4b^ and p21^WAF1/Cip1^ promoters,^90,91^ suggesting the pro-proliferative role of MSC-derived exosomal miRNAs.

Liu and colleagues^83^ observed that exosomal lncRNA KLF3-AS1 derived from human MSCs induced OA chondrocyte proliferation and chondrogenic genes such as type II collagen (COL2A1) and aggrecan but suppressed the expression of MMP13 and its direct upstream transcription factor runt-related transcription factor 2 (RUNX2). The results also demonstrated that exosomal lncRNA KLF3-AS1 acted as an endogenous sponge for miR-206, thus relieving miR-206-mediated suppression of G-protein-coupled receptor kinase interacting protein-1 (GIT1), a scaffold protein that can bridge multiple protein interaction networks to promote chondrocyte proliferation.^92,93^ Knockdown of GIT1 significantly reversed exosomal lncRNA KLF3-AS1-mediated chondrocyte proliferation, highlighting the significance of the exosomal lncRNA KLF3-AS1/miR-206/GIT1 axis in tuning chondrocyte function and OA progression.^83^

Beyond miRNAs and lncRNAs, the crucial role of EV-derived circRNAs in cell proliferation has emerged. Circular RNA homologous domain interacting protein kinase 3 (circHIPK3), produced from exon 2 of the HIPK3 gene, is highly abundant in mammalian cells as well as exosomes shed by them and exhibits multifaceted roles in controlling cell growth across tissue types by sponging diverse miRNAs.^94,95^ Li and colleagues^74^ confirmed that MSC-EV-derived circHIPK3 tended to sponge miR-124-3p, a well-known antiproliferative miRNA, thus remarkably promoting chondrocyte growth and attenuating OA chondrocyte injury. These studies suggest that exosomal ncRNAs may be involved in OA development by regulating chondrocyte proliferation.

#### Exosomal ncRNAs regulate chondrocyte apoptosis in OA

In the late stage of OA, cartilage is characterized by hypocellularity and lacunar emptying due to excessive chondrocyte apoptosis. Chondrocyte apoptosis damages the synthesis and catabolic pathways of ECM contents, which eventually leads to ECM cartilage loss. In turn, the decrease in ECM contents further exacerbates chondrocyte apoptosis, thus entering a vicious cycle of chronic degeneration. Chondrocyte apoptosis can be triggered by multiple factors, including mechanical stress, cytokines (e.g., IL-1β), and reactive oxygen species (ROS).^96^ Several studies have emphasized the involvement of exosomal ncRNAs in these apoptotic pathways.^97–99^

Articular cartilage absorbs mechanical stress to stabilize the joints, constantly exposing chondrocytes to a combination of different forces. High levels of cyclic loading cause chondrocyte death and the extent of the lesion is increased with impact intensity.^100^ In response to cyclic loading, chondrocytes produce aberrant levels of ROS through NADPH oxidase (NOX).^101–103^ Li and colleagues^97^ revealed that human cord mesenchymal stem cell (hucMSC)-derived exosomal miR-100‐5p targeted NOX4 to inhibit cyclic strain‐induced ROS production and apoptosis in primary articular chondrocytes. Moreover, overexpression of NOX4 attenuated the exosomal miR-100-5p-mediated protective effects on chondrocytes. Their study preliminarily demonstrated the potential of hucMSC-derived exosomal miR-100‐5p to eliminate cyclic strain-induced chondrocyte apoptosis.

IL-1β, a proinflammatory cytokine, has been widely reported to induce chondrocyte apoptosis and cartilage destruction and is commonly used to establish in vitro models of OA. IL-1β treatment remarkably alters chondrocyte ncRNA profiles. Wang and colleagues demonstrated that IL-1β elevates lncRNA LYRM4 in chondrocytes, while exosomes originating from human bone MSCs can be taken up by chondrocytes and alleviate IL-1β-responsive LYRM4 levels and cellular apoptosis.^98^ However, which cargo takes control of this process needs more clarification. Similarly, circ-BRWD1 (circ_0116061) and circ_0001846 were increased in the IL-1β-treated chondrocyte cell line CHON-001 and the exosomes shed by these cells, as well as in OA cartilage tissues.^73,99^ Exosomal circ-BRWD1 and circ_0001846 further elevated IL-1β-induced apoptosis and MMP13 levels by eliminating the bona fide function of miR-1277 and miR-149 in CHON-001 cells, respectively. These studies suggest that exosomal ncRNAs functionally integrate into the apoptotic process of chondrocytes to modulate OA progression.

#### Exosomal ncRNAs regulate chondrocyte autophagy in OA

Autophagy is a highly conserved degradation system involving intracellular energy recycling among species and is generally activated by oxidative and metabolic stress.^104^ It serves as a protective mechanism in normal cartilage, while the compromised autophagy level favors OA-like changes in chondrocytes.^105,106^ With the development of OA, the expression of the mammalian target of rapamycin (mTOR), a major negative regulator of mammalian autophagy, is upregulated. A study by Wu and colleagues^77^ demonstrated that infrapatellar fat pad (IPFP) MSC (MSC^IPFP^)-derived exosomes could significantly enhance autophagy levels by inhibiting mTOR, which ultimately rescued IL-induced OA-like gene expression (MMP13 and ADAMTS5) in chondrocytes. Subsequently, exosomal RNA-seq and luciferase reporter assays revealed that miR-100-5p could target the mTOR 3’UTR among the predicted miRNAs. Intra-articular injection of antagomir-100-5p significantly demolished the remedial effect of MSC^IPFP^-Exos on damaged cartilage in a mouse model of surgical destabilization of the medial meniscus (DMM). Therefore, these results suggest the potential of exosomal miRNAs originating from MSC^IPFP^ to protect articular cartilage from OA injury. However, evidence regarding the role of exosomal lncRNAs and circRNAs in OA-related autophagy is still lacking.

### Exosomal ncRNAs and inflammatory microenvironment in OA

OA has been previously considered a “wear disease”.^107,108^ However, cumulative evidence suggests the contribution of chronic low-grade inflammation to OA symptoms and progression.^14,109^ Indeed, degraded ECM components or cartilage fragments accumulate in the articular cavity and are viewed as DAMPs that provoke inflammatory responses in peripheral stromal cells, including FLSs and macrophage-like synoviocytes. As a result, multiple proinflammatory factors, such as cytokines and chemokines, are released into the synovial fluid and in turn aggravate the loss of chondrocyte phenotypic stability and the degradation of ECM, causally creating a microenvironment favoring cartilage lesions. Sustained low-grade inflammation in this microenvironment alters the secretory profile of synovial tissue, particularly exosomal content.^110^ Currently, the engagement of exosomes in maintaining the OA inflammatory network has received extensive scrutiny.

#### Exosome-mediated crosstalk between FLSs and chondrocytes

FLSs, also termed synovial fibroblasts, populate the intimal lining layer of the synovial membrane. They normally secrete joint fluid that lubricates the articular cartilage. In an osteoarthritic state, however, these cells tend to express excessive MMPs and ADAMTS that tip the balance between cartilage synthesis and catabolism towards tissue destruction and behave as inflammatory aggressors through the release of exosomal cargoes, including potentially regulatory miRNAs. NanoString analysis demonstrated that the levels of 50 miRNAs differ between exosomes from IL-1β-stimulated and nonstimulated FLSs.^111^ FLS-derived exosomes can be readily endocytosed by chondrocytes, suggesting tight contact between these cell lineages via exosomes carrying miRNAs. miR-126-3p, a transcriptional modulator of chronic inflammation and innate immunity,^112^ is significantly reduced in synovial exosomes in OA patients,^113^ while treatment of exosomes from FLSs overexpressing miR-126-3p effectively suppresses apoptosis and inflammatory cytokine levels (IL-1β, IL-6, and TNF-α) in rat chondrocytes. Consistently, exosomal miRNA-126-3p exerts antiapoptotic and anti-inflammatory effects on articular cartilage in vivo, indicating that FLSs modulate cartilage inflammatory responses via exosomal miRNAs.

In addition to miRNAs, some exosomal lncRNAs, such as lncRNA-H19^78^ and lncRNA-PCGEM1,^79^ were involved in FLS-chondrocyte communication. H19 was significantly decreased in chondrocytes treated with IL-1β,^78^ whereas overexpressed exogenous H19 in FLSs can be transferred to chondrocytes through exosomes. Exosomal H19 was able to serve as a sponge for miR-106-5p, a well-characterized driver of inflammatory bone loss by targeting tissue inhibitor of metalloproteinase 2 (TIMP2),^114,115^ therefore weakening MMP13 and ADAMTS5 expression in chondrocytes.^78^ Conversely, the upregulation of lncRNA-PCGEM1 was observed in OA patient cartilage and in exosomes isolated from OA FLSs. Exosomal PCGEM1 facilitated IL-1β-induced chondrocyte apoptosis and cartilage matrix degradation.

In a persistent chronic inflammatory environment, FLSs also become passively responding effectors of exosomes shed by chondrocytes, suggesting mutual regulation between FLSs and chondrocytes to promote OA inflammation. When exosomes from IL-1β-treated chondrocytes are applied to OA synovium, a marked elevation in MMP13, IL-1β, TNF-α, and cyclooxygenase-2 (COX-2) was observed compared with nontreated exosomes.^116^ This potential positive feedback loop between FLSs and chondrocytes highlights the role of exosomes in contributing to the complexity of OA inflammatory networks.

#### Role of exosomes in macrophage-involved inflammation

**Fig. 5:**
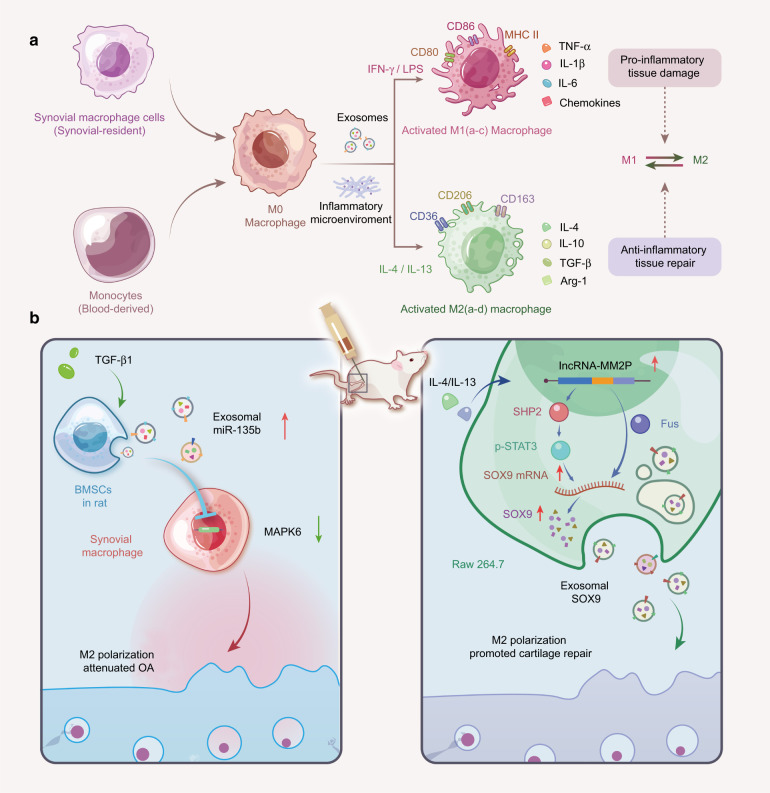
Macrophage polarization in cartilage tissue. **a** The phenotype of macrophage polarization. Synovial macrophages can be derived from monocytes that originally reside in synovial tissue or in the circulation. Evidence suggests that primary macrophages (M0) can be activated and differentiate into the M1 phenotype (INF-γ and LPS stimulation) and M2 phenotype (IL-4 and IL-13 stimulation). Both M1-polarized (subdivided into M1a-c, which promotes the inflammatory response) and M2-polarized (subdivided into M2a-d, which suppresses the inflammatory response) macrophages can regulate the function of cartilage tissue through the secretion of exosomes carrying specific cytokines and chemicals. The conversion of the M2 phenotype into the M1 phenotype can effectively delay OA degeneration. **b** The role of exosomes in regulating macrophage polarization in OA. Left: TGF-β1-stimulated BMSC-derived exosomes attenuate cartilage injury by promoting M2 polarization of synovial macrophages through the miR-135b/MAPK6 signaling pathway. Right: Upon IL-4 or IL-13 stimulation, lncRNA MM2P is upregulated in rat macrophage (RAW264.7) cells and blocks SHP2-mediated dephosphorylation of STAT3 at Try705, thus promoting the transcription of the SOX9 gene. Alternatively, MM2P interacts with the RNA-binding protein FUS to stabilize SOX9 mRNA. Consequently, the elevated SOX9 mRNA and proteins can be delivered to chondrocytes via exosomes to promote cartilage repair. INF-γ interferon-gamma, LPS lipopolysaccharide, IL-4 interleukin (IL)-4, IL-13 interleukin (IL)-13, TGF-β1 transforming growth factor-beta1, BMSCs bone marrow mesenchymal stem cells, MAPK6 mitogen-activated protein kinase 6, SOX9 SRY-box transcription factor 9, STAT3 signal transducer and activator of transcription 3, ncRNAs noncoding RNAs, OA osteoarthritis

FLSs are interdigitated with macrophage-like synoviocytes to constitute a delicate structure of the synovial intima. Aberrant activation or “polarization” of synovial macrophages by cartilage matrix catabolic molecules and cytokines drive synovial inflammation and OA progression.^109,117,118^ The polarization phenotype of macrophages varies greatly depending on the stimuli of the local environment^119^ (Fig. 5). For example, lipopolysaccharide (LPS), interferon-gamma (IFN-γ), or granulocyte-macrophage colony-stimulating factor (GM-CSF) can induce M0 macrophages into an M1 phenotype, producing proinflammatory cytokines (TNF-α, IL-1β, and IL-6) to elicit proinflammatory responses.^118^ In contrast, interleukin-4 (IL-4)/interleukin-13 (IL-13) stimulates M0 macrophages into an M2 phenotype to secrete anti-inflammatory cytokines (IL-10) and growth factors (TGF-β), ultimately suppressing inflammation and promoting tissue repair.^109^ In addition, M1 and M2 macrophages display distinct markers. The M1 phenotype highly expresses membrane receptor cluster differentiation 86 (CD86), toll-like receptor 4 (TLR4), and inducible nitric oxide synthase (iNOS),^120^ while the M2 phenotype is characterized by cluster differentiation 163 (CD163).^119^ The accumulation of M1-polarized macrophages in the synovial intima is the morphological characteristic of synovial inflammation, and the polarization status highly correlates with OA severity.^121^ In this regard, the progression of OA can be alleviated by facilitating the reprogramming of macrophages from the proinflammatory M1 to the anti-inflammatory M2 subset.^122^

The involvement of exosomes in determining macrophage phenotype and behavior in the inflamed OA joint has been highlighted. Exosomes isolated from TGF-β-treated bone MSCs increased the percentage of CD163-positive macrophages (M2) in OA rat tissues. Meanwhile, these exosomes guide the phenotypic switch of LPS-primed macrophages from M1 to M2. Among the exosomal contents, miR-135b was highly expressed and probably actively involved in M2 transition by targeting mitogen-activated protein kinase 6 (MAPK6), thereby alleviating cartilage damage.^123^ Exosomes are also linked to the inflammatory behavior of polarized macrophages. Upon the stimulation of exosomes isolated from the synovial fluid of OA patients, GM-CSF-primed M1 macrophages release abundant IL-1β and chemokines,^124^ indicating that exosomes in synovial fluid serve as a key mediator to create the inflammatory microenvironment in OA. Another study^75^ found that exosome-like vesicles originating from OA chondrocytes can be internalized by LPS-primed macrophages and further enhance inflammasome activation and subsequent IL-1β processing. Mechanistically, these vesicles likely inhibit autophagy-related 4B (ATG4B) expression by delivering its upstream modulator miR-449a-5p into target macrophages. Subsequently, the decreased autophagy promoted the production of mitochondrial ROS, a potential signal to promote IL-1β production, suggesting the proinflammatory role of exosomes released from OA chondrocytes. Instead, exosomes from normal chondrocytes restore mitochondrial dysfunction and trigger the macrophage response towards the M2 phenotype.

Alternatively, macrophages can modulate chondrocyte behavior via exosomes. Upon IL-4 or IL-13 treatment, RAW264.7 murine macrophages and mouse bone marrow-derived macrophages undergo M2 polarization, with a dramatic increase in lncRNA MM2P.^125^ MM2P upregulates the expression of SRY-box transcription factor 9 (SOX9) in M2 macrophages either by interacting with the RNA-binding protein FUS to stabilize SOX9 mRNA or by blocking SHP2-mediated dephosphorylation of signal transducer and activator of transcription 3 (STAT3), which promotes SOX9 transcription. The subsequent elevated SOX9 mRNA and protein can be packaged into exosomes and transferred to mouse chondrocytes, where they can promote the secretion of ECM components. Collectively, mutual crosstalk between macrophages and other cell populations is largely attributed to exosomes released from them.

### Exosomal ncRNAs and subchondral bone remodeling in OA

The articular cartilage, calcified cartilage, and subchondral bone constitute osteochondral units that work in concert to support the functional loading and motion of the joint, while progressive heterogeneous changes in the osteochondral tissues occur during OA evolution.^126^ ECM loss in the OA cartilage layer reduces its ability to absorb mechanical pressure and leads to excessive loading on the subchondral bone. Gradually, however, the fine balance between osteoclast-mediated bone resorption and osteoblast-mediated bone formation is perturbed, bringing about heterogeneity in the subchondral density or stiffness that creates local shear forces to intensify cartilage deformation and further damage.^127,128^ Hence, OA is no longer considered a simple subject bothering articular cartilage but rather an entire joint disorder is concurrently driven by subchondral bone remodeling and subsequent neovascular invasion into the synovium and articular cartilage.^129^

During abnormal subchondral bone remodeling, the formation of microcracks, combined with abnormal vascularization, facilitates molecular transport between cartilage and subchondral bone. In view of this, the crosstalk between the subchondral and cartilage layers may be elevated in OA.^130^ Subchondral osteocytes can induce phenotypic changes in OA chondrocytes.^131^ This interaction might be conducted in an exosome-dependent fashion.^132,133^ For example, Wu et al isolated and purified exosomes from osteoblasts in the non-sclerotic or sclerotic regions of human OA subchondral bone. After co-culture with chondrocytes, exosomes from sclerotic osteoblasts triggered the expression of chondrocyte catabolism genes and reduced the expression of chondrocyte-specific markers. RNA-seq confirmed the high enrichment of miR-210-5p in the exosomes of OA sclerotic osteoblasts. Exosomal miR-210-5p increased hypertrophic and ECM-degradative gene expression and inhibited cellular aerobic respiration in chondrocytes, a feature frequently observed in OA conditions.^134^ Another study demonstrated a compelling case of pathological exosomal miRNA communication between osteoclasts and neighboring chondrocytes in OA.^135^ Following anterior cruciate ligament transection (ACLT) surgery to establish a mouse OA model, a set of miRNAs (miR-21a-5p, miR-214-3p, miR-30a-5p, and miR-30d-5p) were elevated in bone marrow osteoclasts, as well as in exosomes shed from them. Nevertheless, ablating Dicer, which is essential for miRNA biogenesis, or silencing Rab27a, a GTPase that controls exosome secretion, remarkably delayed OA progression. Strikingly, upon intravenous injection of osteoclast-derived exosomes, the preferential homing of these vesicles to chondrocytes was observed in ALCT cartilage, supporting the presumption that osteoclast-derived exosomes may pass through microcracks and vascular channels to cartilage during OA evolution. Among the exosomal contents, miR-214 was demonstrated to blunt TIMP2/3, the endogenous antagonists of MMPs and ADAMTS, consequently promoting ECM catabolism and cartilage degeneration. These studies suggest that the connection between chondrocytes and subchondral osteocytes is enhanced by exosomal miRNAs, which appear to accelerate OA progression.

## Exosomal proteins in OA

Previous proteomic studies have found that proteins in exosomes are involved in OA progression.^136,137^ For example, Zheng and colleagues^138^ analyzed the protein composition of exosomes from mouse primary normal chondrocytes (D0) and IL-1β-treated chondrocytes (D3) and identified 2409 and 2077 proteins in the D0 and D3 exosomes, respectively. Subsequently, Ingenuity Pathway Analysis (IPA) and Gene Ontology (GO) analysis were performed among these identified proteins and revealed that the proteomic pattern of D3 exosomes was similar to that of D0 exosomes. However, the number of identified mitochondrial proteins was decreased in D3 exosomes, indicating the damaged chondrocyte mitochondria upon IL-1β stimulation. More importantly, intra-articular administration of D0 exosomes restored mitochondrial dysfunction and polarized the macrophage response to the M2 phenotype, successfully preventing the development of OA in mice, suggesting a potential role of chondrocyte exosomal proteins in tuning mitochondrial function. Gao and colleagues^139^ demonstrated that exosomes derived from knee joint fluid of osteoarthritis patients expressed significantly higher levels of inflammatory cytokines (e.g., IL-1β, TNF-α, and IFN-γ) in end-stage OA patients than in early-stage OA patients, which favored inflammation and cartilage degeneration during OA progression. In addition, Zhang and colleagues^140^ reported that exosomes from human embryonic stem cell-derived MSCs were able to express CD73, which can efficiently activate AKT and ERK signaling to enhance chondrocyte proliferation and infiltration, while the administration of adenosine 5′-(α,β-methylene) diphosphate (AMPCP), a CD73 inhibitor and theophylline, an adenosine receptor antagonist, effectively reduced MSC exosome-induced AKT and ERK phosphorylation in chondrocytes, highlighting the key role of exosomal CD73/adenosine axis during exosome-mediated cartilage repair. To date, there is limited evidence characterizing OA-associated exosomal proteins and the role of exosomal proteins in OA can be further explored.

## Clinical significance of exosomes in OA

### Exosomal cargoes as potential biomarkers for OA

Currently, X-ray^141^ and magnetic resonance imaging (MRI)^142^ are the so-called “gold standard” for OA diagnosis. When imaging signs occur, however, the degeneration of articular cartilage has often progressed to the middle and late stages that require active intervention and treatment. Although previous studies have shown the potential of matrix metalloproteinases (MMPs), lipopolysaccharide-binding protein (LBP), soluble toll-like receptor 4 (sTLR4), and urinary C-telopeptide of type II collagen (uCTX-II) as biomarkers for OA,^143–145^ but further work is required to validate their effectiveness. Hence, OA is now acknowledged as a whole joint disorder featuring heterogeneous variations in articular tissues, but the current strategies to detect such changes are unavailable due to the lack of reliable markers that indicate early changes and ongoing disease pathophysiology over time or reflect the relative involvement among individual joint tissues.

Exosomes carrying a wide range of ncRNAs have been emphasized as potential biomarkers in a variety of diseases,^146–150^ benefiting from their high abundance in body fluids,^151,152^ as well as the unique expression signatures of their cargoes as the disease evolves. Of note, the double-lipid membrane protects their cargo from enzyme-mediated degradation or clearance by immune systems, endowing them with an extended half-life compared with the free molecules and prompting the attention to evaluate their clinical feasibility for given diseases.^137^ In OA joint disease, the disruption of synovial tissue homeostasis results in an altered secretory exosome profile, which could potentially reflect the complexity and heterogeneity of the disease. Kolhe and colleagues^43^ characterized exosomal miRNAs in synovial fluids from OA patients and showed that the miRNA profiles differed between male and female OA patients. Certain miRNAs specific to female OA patients are relevant to the estrogen response and Toll-like receptor (TLR) signaling. For example, miR-26a, an inflammatory suppressor that targets Toll-like receptor-3 (TLR3) to ameliorate arthritis severity,^153^ can be induced by estrogen in FLSs, while its downregulation is observed in exosomes from female OA patients, which possibly provides hints to explain the increased prevalence and severity of OA in females than in males. Meanwhile, their team also illustrated sex-specific differences in exosomal protein cargo in synovial fluid from OA patients.^154^ These findings may contribute to the development of exosomal cargo-guided sex-specific monitoring for OA.

Synovial fluid-derived exosomes also vary in their contents in different OA stages. A recent study^155^ suggested the value of synovial fluid-derived exosomal lncRNA-PCGEM1 in discriminating the early and advanced stages of OA. Exosomal PCGEM1 in synovial fluid was significantly elevated in late OA compared with early OA, and its potential to distinguish early and late OA has been suggested based on the area under the receiver operating characteristic curve (AUC).

Circulating ncRNAs, especially miRNAs that mark early- and advanced-stage OA, have been evidenced.^156,157^ However, the primary concern regarding the various types of circulating biomarkers is their specificity solely for OA or at least their ability to reflect the main characteristics of cartilage degeneration. Comprehensive investigations on the clinical significance of circulating exosomal ncRNAs in the diagnosis and tracking staging of OA are still lacking. Future studies may aim to address the feasibility of examining joint tissue-derived exosomes in blood samples and discriminating the cellular origin of exosomes, which may open up the possibility to sense tissue-specific alterations as indicators for OA in a minimally invasive manner.

### Potential therapeutic strategies based on exosomes

**Fig. 6:**
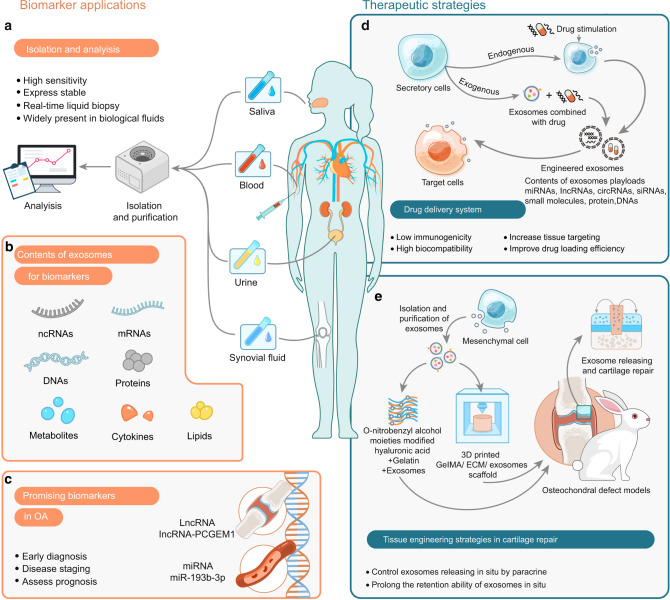
The potential clinical application of exosomes in OA. **a** Isolation and analysis of exosomes. Pipeline of exosome separation, purification, and identification. **b** Exosomal contents for biomarkers. Exosomes are rich in bioactive molecules, including DNAs, mRNAs, ncRNAs, proteins, metabolites, cytokines, and lipids, and can be used as potential biomarkers; **c** Exosomal ncRNAs as biomarkers for OA. Exosomal miRNA and lncRNA can be used for diagnosis, prognosis, and monitoring of OA. **d** Exosomes act as a drug delivery system in OA. Exosomes are isolated from naive or modified donor cells to promote the efficiency of cargo delivery. **e** Exosome-based tissue engineering strategy to repair cartilage. Exosomes engineered for cartilage repair, combined with hydrogel scaffolding and 3D printing techniques, effectively promote in situ repair of cartilage defects. OA osteoarthritis, ECM extracellular matrix

Improved understanding of exosomes in OA pathophysiology enables the development of potential therapeutic approaches to block or limit joint structure destruction. Exosome-based vehicle delivery systems and tissue engineering techniques represent advanced therapeutic strategies that have recently attracted increasing attention (Fig. 6).

#### As a promising therapeutic delivery system in OA therapy

MSCs exhibit excellent performance in tissue repair and regeneration and serve as promising seed cells in cartilage engineering approaches. These advantages are at least partially based on the paracrine effects of cellular exosomes that carry various ncRNAs to promote proliferation and inhibit inflammation in recipient cells. Therefore, MSC-derived exosomes have been considered a promising delivery system for OA therapy.

Recently, several studies have attempted to use naive MSC-derived exosomes to treat OA in vivo and in vitro models. Two individual studies demonstrated that bone marrow MSC-derived exosomes display a beneficial therapeutic effect on OA by transferring regulatory miRNAs to cartilage and balancing the synthesis and degradation of ECM.^158,159^ However, they focused on different miRNAs in bone marrow MSC-derived exosomes, and which cargo dominates the delay of cartilage degeneration remains unclear. To address this, some studies manipulate donor MSCs by virtue of transfection with genes of interest. miR-140, one of the most important miRNAs in chondrocytes, plays a central role in both cartilage development and homeostasis.^160^ Tao et al.^76^ overexpressed miR-140-5p in synovial MSCs using a lentiviral system and showed consistently elevated levels of miR-140-5p in the exosomes shed by these cells. Compared with non-modified exosomes, miR-140-5p-abundant exosomes showed an enhanced ability to trigger chondrocyte proliferation and migration without hindering ECM secretion in vitro and successfully prevented cartilage degeneration in OA rat models. Similarly, transfection of dental pulp stem cells (DPSCs) with miR-140-5p mimics enriches miR-140-5p in their exosomes, and administration of these exosomes remarkably improves joint conditions in a rat OA model.^161^

In addition to the genetic manipulation of exosomal cargoes, targeted modification of exosomes also serves as a promising OA treatment approach to deliver cargoes into chondrocytes across the dense and nonvascular ECM.^162^ To this end, dendritic cells are transfected with plasmids encoding fusing protein of chondrocyte-affinity peptide (CAP) and lysosome-associated membrane glycoprotein 2b (Lamp2b), which allows the release of exosomes displaying CAP-Lamp2b on the surface. The engineered exosomes are subsequently loaded with miR-140 via electroporation and are capable of transferring miR-140 into deep cartilage regions, thus alleviating OA progression in a rat model.^162^ These findings may allow targeted regulation of intercellular communication and point toward a potential cell-free therapy of OA alternatives to cell-based MSC therapy.

Certainly enhanced cargo delivery conducted by exosomes hinders cartilage homeostasis, which may be blocked in OA therapy. Nevertheless, the biogenesis of exosomes relies on normal cellular processes. Disrupting their activity in specific cell types or tissues remains challenging. Indeed, preferential suppression of exosomal ncRNA crosstalk to manipulate physiological or pathological processes has not yet been evidenced in vivo.

#### Tissue engineering strategies based on exosomes in OA therapy

Although evidence of penetrance of exosomal cargoes into cartilage is compelling, their persistence and efficacy must be carefully examined to optimize clinical potency. Tissue engineering techniques, such as hydrogels and scaffolds, have been used to better locate exosomes for exogenous drug delivery at specific cartilage defects. With these supporting materials, the retention of exosome cargo at locally defective sites can be increased to improve therapeutic efficacy.^163,164^ Liu and colleagues^163^ developed a photoinduced imine crosslinking hydrogel glue as an exosome scaffold to prepare an acellular tissue patch (EHG) for cartilage regeneration. The results showed that the EHG tissue patch could integrate seamlessly with natural cartilage, effectively preserving exosomes at defective sites and promoting cartilage repair and regeneration. Recently, three-dimensional (3D) printing technology has also been applied to the treatment of OA. Chen and colleagues^164^ designed a bioscaffold for MSC exosome delivery and fabricated a 3D printed ECM/gelatin methacrylate (GelMA)/exosome scaffold, which effectively restored chondrocyte homeostasis, polarized synovial macrophages to the M2 phenotype, and significantly promoted cartilage regeneration in animal models. Such properties add the possibility of exosomes for utility within regenerative therapies for OA.

## Conclusions and prospects

Exosome represents a novel mode of cell-to-cell communication in joint tissues. Exosomal contents, especially ncRNAs, are selectively packaged and transferred to recipient cells to regulate their biological behaviors. Certain exosomal ncRNAs showed different expression patterns between the synovial fluid of OA patients and healthy controls, making them signs of disease onset and promising biomarkers that may inform inflammation types and disease status. Accumulating evidence also highlights novel therapeutic strategies based on exosomal ncRNAs, some of which have shown promising outcomes in vivo.

Despite the recent progress made in characterizing exosomes in OA, some challenges remain unresolved in this field, particularly in terms of the identification of the cellular origin of exosomes. Indeed, exosomes in body fluids are often heterogeneous vesicles of various origins, making it difficult to identify and isolate joint cell-specific exosomes in OA samples. Next, the involvement of exosomes in cell-to-cell communication is achieved by the delivery of specific bioactive molecules. However, how these molecules are selectively packaged into exosomes is unclear, and further studies are needed to reveal detailed mechanisms underlying exosome biogenesis. To date, only a small fraction of exosomal cargo has been identified in OA, most of which are ncRNAs. How other exosomal contents function in OA requires further investigation.

OA pathology is marked by abnormal regulation of gene expression in multiple ways. Nevertheless, a considerable portion of exosomal studies have focused merely on cartilage, synovium, or subchondral bone in isolation, ignoring the fact that OA is a whole joint disorder. In the case of viewing these in vitro studies, however, caution must be taken in regard to the pathophysiological correlation, such as whether the stimuli can pathologically mimic the inducers of arthritis. Additionally, the field to examine the therapeutic potential of exosomal ncRNAs is still in its infancy. Current studies confirm that exosomes can be engineered to deliver therapeutic factors to recipient cells for therapeutic applications, but one premise is that exosomes must be able to accurately find their targets and release their cargo to target the specificity of tissues such as chondrocytes and synovial cells. Hence, more studies are needed to identify specific markers on the membrane of joint cell populations. Accordingly, exosomes with surface modifications are designed to identify these markers, enabling targeted intervention of intercellular crosstalk. Moreover, OA is a chronic degenerative disease and the progression of OA can be controlled using “stepped therapy” at different stages. Therefore, how to accurately subtype OA by exosomes, especially in the early stage of OA, is crucial for the clinical treatment of OA. In addition, previous studies have evaluated the feasibility of sampling patient blood or synovial fluid to isolate exosomes for OA diagnosis. Non-invasive methods for exosome acquisition from other body fluids (e.g., urine, saliva) are expected to relieve the pain of patients. With an increasing exploration of these unknowns, we believe that exosomes will become feasible in the future diagnosis and treatment of OA.
